# Bovine xanthine oxidase electrocatalysis: substrate oxidation and its role in nitrite reduction

**DOI:** 10.1007/s00775-026-02146-z

**Published:** 2026-04-27

**Authors:** Peter D. Giang, Dimitri Niks, Russ Hille, Paul V. Bernhardt

**Affiliations:** 1 School of Chemistry and Molecular Biosciences, University of Queensland, Brisbane, 4072 Australia; 2 Department of Biochemistry, University of California, Riverside, CA 92521 USA

**Keywords:** Molybdenum, Electrochemistry, Catalysis, Voltammetry, Nitrite

## Abstract

Xanthine oxidase plays a central role in purine catabolism in animals, plants and bacteria where it oxidises hypoxanthine to xanthine and xanthine to uric acid. In this work we have immobilised bovine xanthine oxidase on a glassy carbon electrode within a glutaraldehyde film and demonstrated hypoxanthine and xanthine oxidase electrocatalysis using mediator methylene blue as a mediator. The role of xanthine oxidase in the reduction of nitrite to nitric oxide has been probed in two ways. Using the electrochemically reduced mediator methyl viologen, nitrite reduction by xanthine oxidase is complicated by non-enzymatic reduction of nitrite by the mediator. Using hypoxanthine as reductant, nitrite reduction occurs but the product nitric oxide rapidly inactivates the enzyme. Xanthine oxidase can be restored to its functional form by redox cycling on an electrode in the presence of sulfide showing that nitric oxide leads to desulfuration of the molybdenum active site.

**Graphical abstract:**

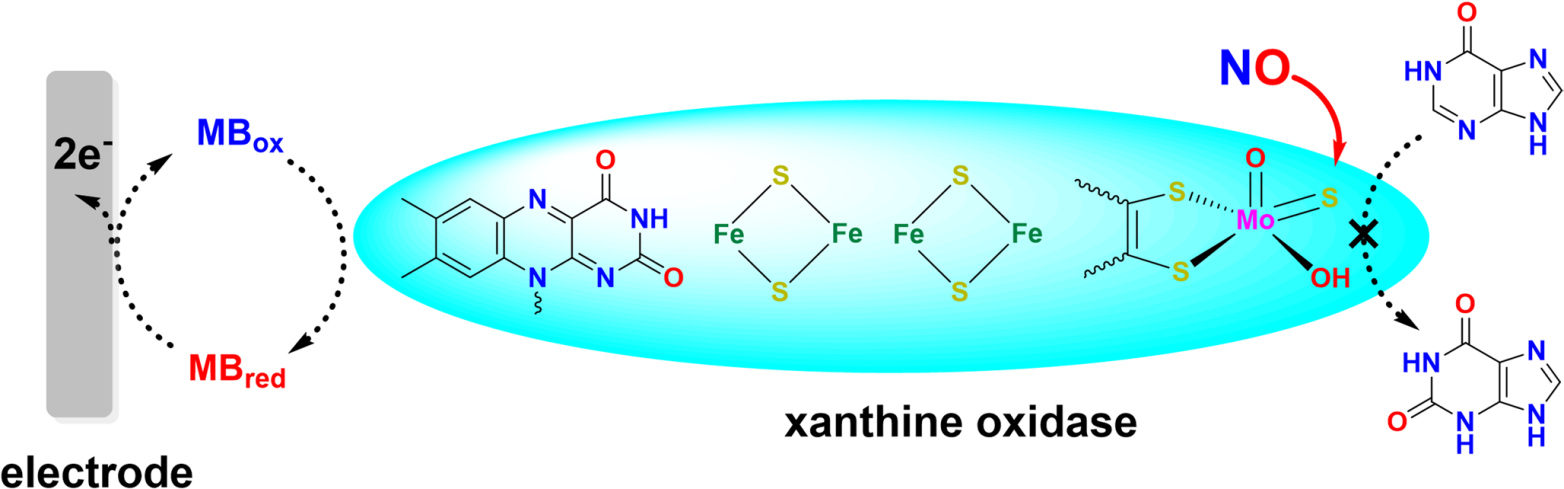

**Supplementary Information:**

The online version contains supplementary material available at 10.1007/s00775-026-02146-z.

## Introduction

The first example of a xanthine oxidoreductase enzyme was isolated from cows’ milk more than a century ago [1, 2]. However, its molybdenum-(Mo)-dependence [3], structural [4] and functional [5] complexity, and its broad distribution in animals, plants and bacteria was not appreciated until much later. A recent historical review of this venerable enzyme has been published [6]. As one of the most studied Mo-containing enzyme systems, xanthine oxidoreductase (either as an oxidase (XO) utilizing O_2_ as oxidizing substrate or a dehydrogenase (XDH) utilizing NAD^+^ instead) catalyses the oxidation of hypoxanthine to xanthine and xanthine to uric acid as the final two steps of purine catabolism. Apart from these two reactions, which are carried out with equally high efficiency (*K*_M_ < 10 µM, *k*_cat_ > 10 s^–1^), other roles for XO have been investigated including as a source of H_2_O_2_ and superoxide (O_2_^–^) as products of dioxygen reduction [7].

The single-electron reduction of nitrite (NO_2_^–^) to nitric oxide (NO) is of considerable biological interest due to the now well-understood signalling role of NO in several physiological processes, including blood vessel dilation [8], neurotransmission [9], inflammation [10], apoptosis [11] and infection [12]. Unlike bacteria, which express dedicated nitrite reductases that generate NO from NO_2_^–^, there is no established reductive pathway for NO production in animals or any other eukaryotes. The only recognised eukaryotic enzymatic pathway for NO production is via an oxidative process involving nitric oxide synthase enzymes which require molecular oxygen and arginine as co-substrates. Under ischemic (reduced blood flow and low oxygen) conditions where nitric oxide synthase is unable to function, it has been hypothesised that the four Mo-dependent enzymes common to all animals (xanthine oxidase [13], sulfite oxidase [14], aldehyde oxidase [15] and the mitochondrial amidoxime reducing component [16]) are able to act as surrogate nitrite reductases to produce NO as a protective agent against tissue damage. Although recent studies have provided strong evidence that sulfite oxidase can catalyse the reduction of NO_2_^–^ to NO under physiological conditions [17] and supported by electrochemical studies [18], the role of the other molybdenum-containing enzymes remains controversial. Biochemical data for XO show a low affinity for nitrite with *K*_M, nitrite_ values in the millimolar range, although there is no consensus even on this [13, 19–22]. Furthermore, non-enzymatic pathways for nitrite reduction to NO in tissues must be also recognised [23].

**Fig. 1:**
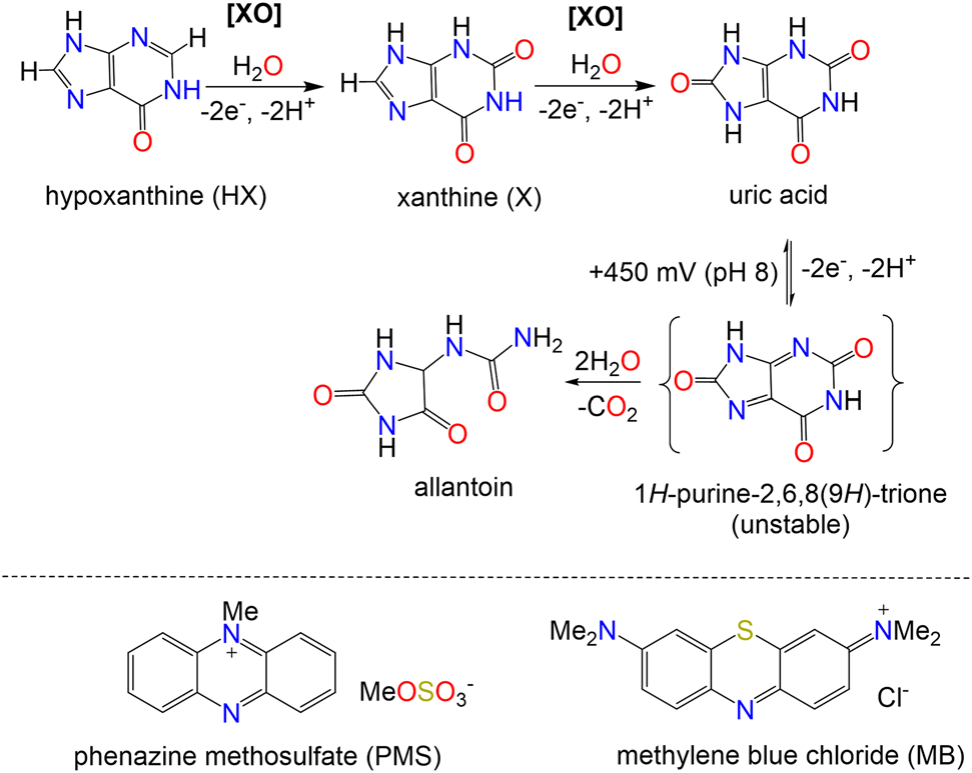
Redox reactions catalysed by xanthine oxidase and other downstream reactions of relevance. Redox mediators relevant to this work are also shown

We have previously used different electrodes and approaches to study the electrochemical properties of bovine XO and bacterial XDH [24–27]. One complication encountered with the anodic electrochemistry of the XO/xanthine or XDH/xanthine systems is that the product (uric acid) is electrochemically active and its 2 electron-oxidised form can mediate enzyme turnover in a rather complicated autocatalytic mechanism [26]. Although the XO-catalysed oxidation of hypoxanthine to xanthine and xanthine to uric acid (Fig. 1) is now well understood [5], the electrochemistry of uric acid is complicated and various unstable intermediates have been observed or proposed that ultimately lead to allantoin as a stable product at neutral pH [28, 29].

In this work we have used electrochemical methods incorporating XO immobilised on the surface of a glassy carbon working electrode to explore both native xanthine/hypoxanthine oxidation and its possible role in nitrite reduction.

## Materials and methods

### Enzyme preparation and reagents

Xanthine oxidase from cow’s milk was purified as described [30]. All other reagents were of analytical grade purity. All solutions were prepared with ultrapure water (resistivity 18.2 MΩ·cm).

### Electrode cleaning and preparation

A three-electrode system was utilised comprising a glassy carbon disk electrode, platinum wire counter electrode, and Ag/AgCl reference electrode (calibrated with quinhydrone, *E*’ = +285 mV vs SHE at pH 7). The electroactive surface area of the glassy carbon electrode was calculated from the cyclic voltammogram of 1 mM ferrocene methanol in 0.1 M KCl solution at multiple scan rates using the Randles-Sevcik equation [31] (Eq. 1, for T = 25 °C). The diffusion coefficient (*D*) of ferrocene methanol is 6.7 × 10^− 6^ cm^2^ s^− 1^ [32], *i*_*p*_ is the current maximum, *n* is the number of electrons (here *n* = 1), *C* is the concentration of analyte (mol cm^–3^), and *υ* is the sweep rate (V s^− 1^). The surface area was determined to be *A* = 0.056 cm^2^.

1 $$\:{i}_{p}=\:2.69\:\times\:\:{10}^{5}{D}^{1/2}{n}^{3/2}AC{\upsilon\:}^{1/2}\:$$

### Electrochemical measurements

Cyclic voltammetry (CV) experiments were conducted at 25 °C with either a Bioanalytical Systems BAS100B/W or BASi EC Epsilon EClipse potentiostat. The glassy carbon electrode was polished mechanically in an aqueous slurry of Al_2_O_3_ (0.05 μm) on a microfibre polishing pad. The electrode was then rinsed and sonicated in water for 15 min and dried in a stream of nitrogen. The electrode was inverted and a premixed solution of XO (3 µL, 90 µM) and aqueous glutaraldehyde (0.25% v/v, 2 µL) was carefully added to the conducting surface. The electrode was placed in a refrigerator (4 °C) for 1–2 h by which time the droplet had dried to a film.

The cell contained 3 mL of solution in HEPES buffer (pH 7.0, 200 mM) for most experiments unless otherwise indicated. All measurements were carried out within a Belle Technology anaerobic glovebox ([O_2_] < 20 ppm).

## Results and discussion

### Electrocatalytic xanthine oxidation

**Fig. 2:**
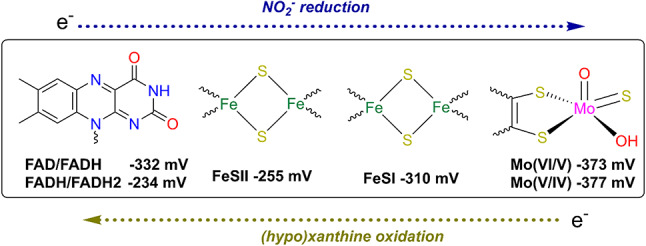
Electron flow during xanthine oxidation and nitrite reduction

The six redox potentials of bovine XO span a relatively narrow range (− 377 to − 234 mV vs. SHE at pH 7.7) [33] as shown in Fig. 2. The FAD cofactor exhibits the highest (most positive) potential and is the site of electron egress following purine oxidation. Therefore, mediators with higher redox potentials are plausible artificial electron acceptors if they exhibit facile electrochemistry and can access the FAD cofactor.

As mentioned above, oxidised uric acid can mediate XO/XDH catalysis [26], so the mediator redox potential should be less than + 450 mV vs. SHE to avoid uric acid interference but high enough to rapidly oxidise the FAD cofactor, the site of electron egress after (hypo)xanthine oxidation. The heterocycle N-methylphenazinum methosulfate (Fig. 1) is suitable in this regard [26, 27, 34] although the instability of this compound in solution is problematic and well documented [35, 36]. Methylene blue chloride (Fig. 1) was used as an artificial electron acceptor for the bovine milk xanthine oxidase/xanthine assay in its earliest investigations [1, 2] and exhibits excellent long term stability so it was used for most experiments in the present study.

**Fig. 3:**
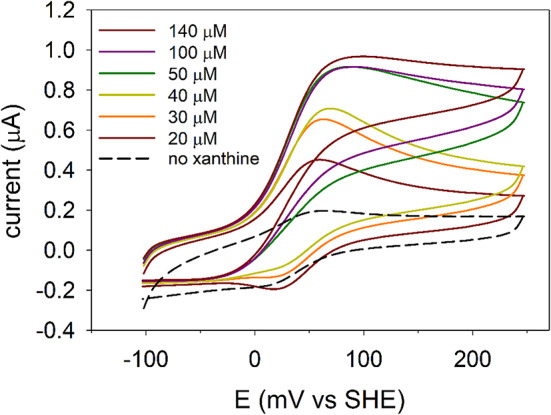
CVs of methylene blue (10 µM) at the glassy carbon/XO/glutaraldehyde electrode in the presence (solid lines) and absence (broken line) of xanthine. Scan rate 5 mV s^–1^, pH 7

The CV of methylene blue at the glassy carbon/XO/glutaraldehyde electrode (Fig. 3, broken curve) exhibits the expected reversible redox response centred at + 40 mV vs. SHE (pH 7). The peak-to-peak separation (ca. 40 mV) is indicative of a 2-electron redox reaction. Upon gradual addition of xanthine to the cell, the anodic current increased and the cathode peak vanished. Quantitative kinetic analysis of the CV profiles requires consideration of electron transfer rates between methylene blue and XO, XO and xanthine as well as substrate diffusion. The peak-shaped profiles seen in the range 20 µM < [xanthine] < 100 µM are again attributed to mass transport limits where xanthine is depleted near the electrode surface resulting in a time/potential-dependent decline in the catalytic current. At [xanthine] > 180 µM the current is no longer limited by xanthine diffusion and remains constant. The concentrations of xanthine in Fig. 3 are all well in excess of the published value of *K*_M, xanthine_ = 3.4(2) µM [37], which highlights how substrate mass transport limits the kinetics rather than the enzyme turnover reaction.

Similar experiments were run using phenazinium methosulfate as the mediator (Supporting Information Figure S1). In that case the higher redox potential of the phenazinium cation (ca. +100 mV vs. SHE) results in a qualitatively faster reaction between reduced XO and the mediator and more rapid depletion of the substrate. In this system much higher xanthine concentrations (> 300 µM) were required to reach a steady state that is no longer limited by mass transport. As mentioned above, the greater stability of methylene blue led to its preferred use in all other experiments.

### Electrocatalytic hypoxanthine oxidation

**Fig. 4:**
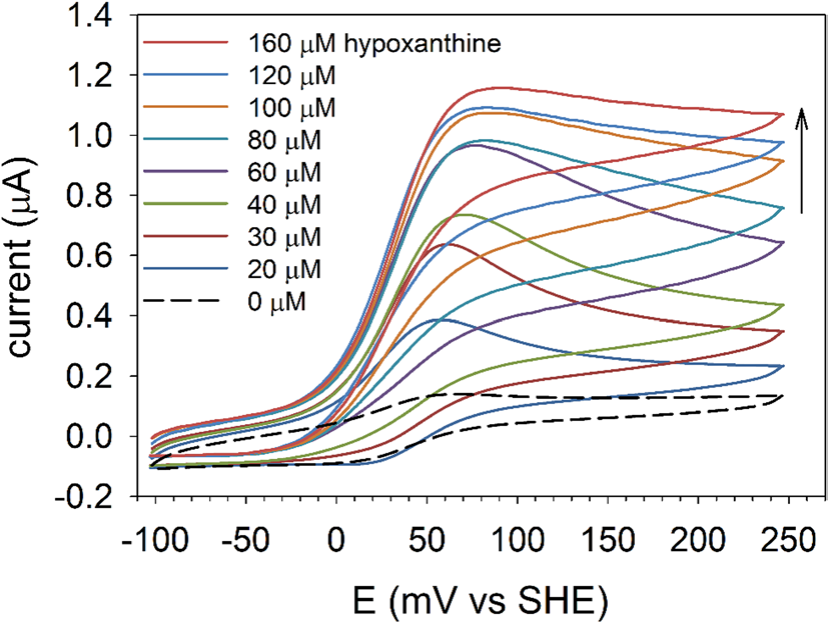
CVs of methylene blue (10 µM) at the glassy carbon/XO/glutaraldehyde electrode in the presence (solid lines) and absence (broken line) of hypoxanthine (HX). Scan rate 5 mV s^–1^, pH 7

When hypoxanthine is the substrate, the product (xanthine) is electrochemically inert within the experimental potential range of − 100 to + 250 mV vs. SHE [27, 28], which averts any product (uric acid)-mediated electrocatalysis. Essentially the same electrochemical behaviour at the glassy carbon/XO/glutaraldehyde electrode is seen upon addition of hypoxanthine to the methylene blue solution (Fig. 4). At low concentrations of hypoxanthine, the CV profile is asymmetric and peak-shaped due to depletion of substrate near the electrode surface. The reported very low *K*_M, hypoxanthine_ = 1.9(1) µM [37] is well below the solution concentrations used so once more the transition from peak shaped to sigmoidal CV profile merely reflects hypoxanthine mass transport limitations.

### Nitrite reduction

For XO-catalysed NO_2_^–^ reduction to be possible, the molybdenum active site must be in its fully reduced Mo^IV^ form, which requires a very low potential electron transfer mediator. Another requirement is that electron flow be reversed compared with native (hypo)xanthine oxidation catalysis (Fig. 2). Both requirements place strict limitations on this system. Relatively few redox mediators have potentials low enough to fully reduce XO. A good candidate is methyl viologen (MV^2+/+^, *E*’ − 430 mV vs SHE) which has been used extensively in electrochemical studies of enzymes due to its low potential and reversible, one-electron redox couple.

**Fig. 5:**
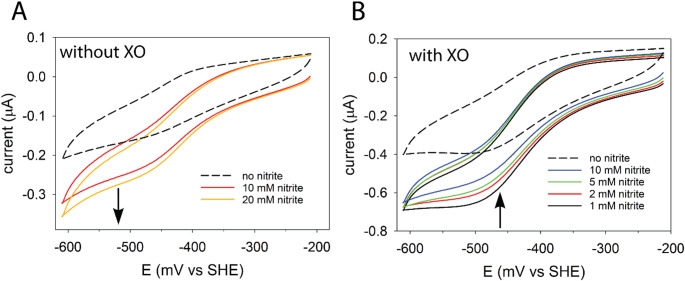
(**A**) CVs of methyl viologen (20 µM) at the glassy carbon/glutaraldehyde electrode (no XO) in the absence (broken curve) and presence of 10 mM nitrite (red) and 20 mM nitrite (yellow) and (**B**) CVs of methyl viologen (20 µM) at the glassy carbon/XO/glutaraldehyde electrode in the absence (broken curve) and presence of nitrite (1–10 mM). Scan rate 5 mV s^–1^, pH 7

However, non-enzymatic electrochemical NO_2_^–^ reduction must also be considered. In the absence of XO, the CV of methyl viologen at pH 7 at a glassy carbon electrode is shown in Fig. 5A (broken curve). The expected reversible response is seen with a midpoint potential around − 420 mV vs. SHE. In the presence of NO_2_^–^ (10 and 20 mM), there is an enhancement of cathodic current. In the absence of methyl viologen, no significant nitrite reduction current in this potential window is observed (data not shown). The data show two important features. Their waveforms in the presence of 10 and 20 mM NO_2_^–^ are sigmoidal and their amplitudes are enhanced, consistent with an electrocatalytic reaction (see Figs. 3 and 4 for similar behaviour). At pH 6.5 (Supporting Information Figure S2) methyl viologen-mediated catalytic NO_2_^–^ reduction was even more pronounced while at pH 8 this effect was small. The observed increase in catalytic current as the pH is lowered is consistent with previously published studies on NO production involving molybdenum enzymes [15, 16, 21, 38]. The conclusion from this control experiment is that (non-enzymatic) reduction of NO_2_^–^ catalysed by methyl viologen in its one-electron reduced form (MV_red_^+^) cannot be ignored and becomes more significant at acidic pH values, as summarised in the following equations:

2a $${\mathrm{MV}}_{{{\mathrm{ox}}}} ^{{{\mathrm{2}} + }} + {\text{ e}}^{{-}} \rightleftharpoons {\mathrm{MV}}_{{{\mathrm{red}}}} ^{ + }$$

2b $${\mathrm{MV}}_{{{\mathrm{red}}}} ^{ + } + {\mathrm{NO}}_{{\mathrm{2}}} ^{{-}} ~ + {\text{ 2H}}^{ + } \to {\mathrm{MV}}_{{{\mathrm{ox}}}} ^{{{\mathrm{2}} + }} + {\text{ NO}} + {\text{ H}}_{{\mathrm{2}}} {\mathrm{O}}$$

Overall

2c $${\mathrm{NO}}_{{\mathrm{2}}} ^{{-}} ~ + {\text{ 2H}}^{ + } + {\text{ e}}^{{-}} \to {\mathrm{NO}} + {\text{ H}}_{{\mathrm{2}}} {\mathrm{O}}$$

The same methyl viologen CV experiments were repeated with the glassy carbon/XO/glutaraldehyde electrode, and the results (at pH 7) are presented in Fig. 5B. Compared to the reversible CV of methyl viologen alone (broken curve), addition of 1 mM NO_2_^–^ results in a significant increase in cathodic current and the waveform takes a sigmoidal shape. Bearing in mind the control data in Fig. 5A, this could simply be interpreted as methyl viologen-catalysed NO_2_^–^ reduction. However, further additions of NO_2_^–^ lead to a monotonic *decrease* in catalytic current which cannot be explained in the same way as the data in Fig. 5A. Similar behaviour is seen at pH 6.5 (Supporting Information Figure S3). This behaviour is indicative of both non-enzymatic and XO-catalysed nitrite reduction, where XO-catalysed NO_2_^–^ reduction decreases with increasing nitrite concentration. This is not due to (reversible) substrate inhibition as (hypo)xanthine oxidase electrocatalytic activity is not recovered with this electrode when fresh buffer, methylene blue and (hypo)xanthine are introduced. Further discussion will be deferred until the following subsection.

**Fig. 6:**
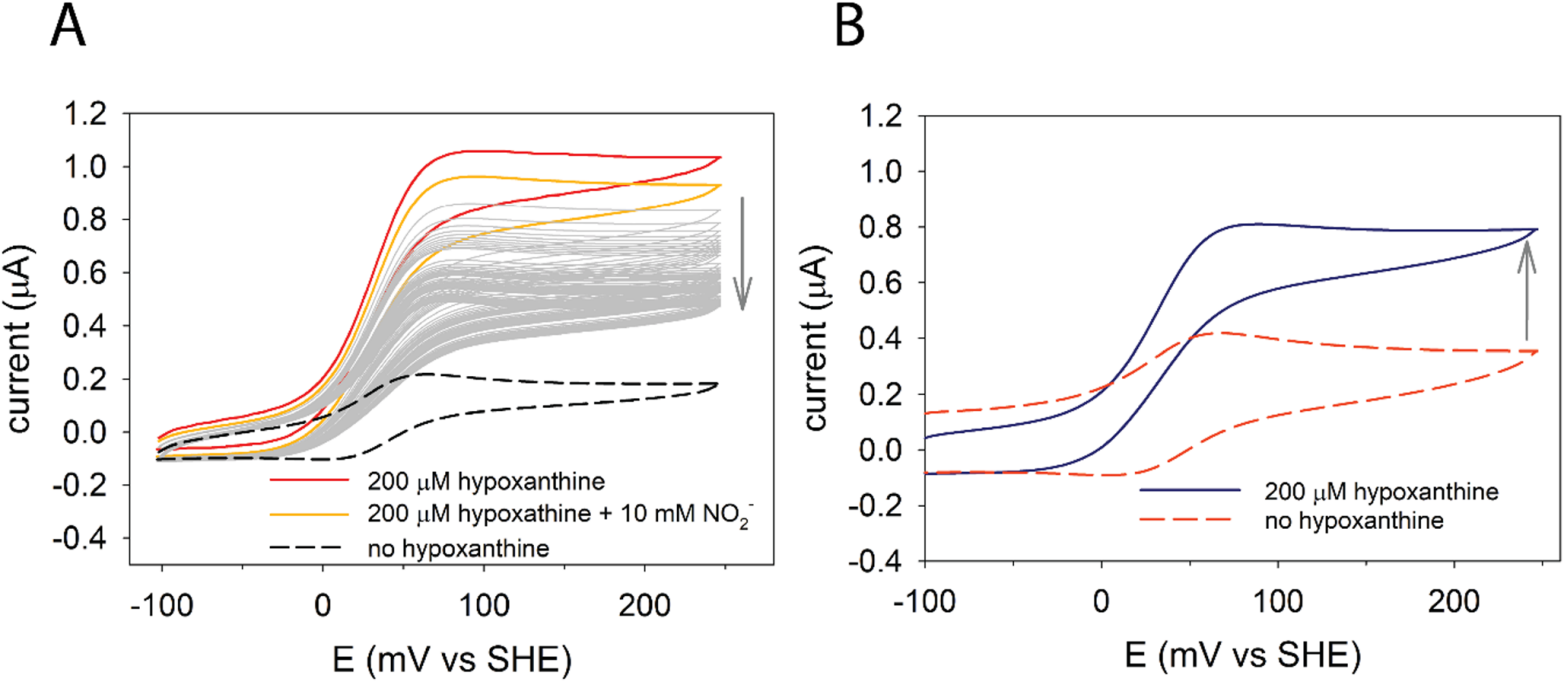
(**A**) CVs of methylene blue (10 µM) at the glassy carbon/XO/glutaraldehyde electrode (no XO) in the absence (broken curve) and presence of hypoxanthine (HX, 200 µM, red curve). The yellow curve is when 10 mM nitrite is added and the grey curves are for successive cycles over about 40 min; (**B**) CV of the same glassy carbon/XO/glutaraldehyde electrode redox cycled with methyl viologen and Na_2_S (see text for details) in the presence of methylene blue (10 µM, broken curve) and with hypoxanthine (200 µM, blue curve), which recovered 80% of original catalytic activity. Scan rate 5 mV s^–1^, pH 7

### Coupled xanthine oxidation and nitrite reduction

Xanthine and hypoxanthine are the only physiological reductants of XO, so if XO acts as a nitrite reductase in vivo then it must be with (hypo)xanthine as the electron donor. NADH (*E*_m,7_ − 320 mV vs. SHE) may also be considered as a reductant of the enzyme, although the reaction takes place at the FAD and back electron transfer to the molybdenum centre is thermodynamically unfavorable (by some 90 mV for the two-electron process, see Fig. 2). The tandem nitrite reduction/(hypo)xanthine oxidation process is demanding in several respects as intramolecular electron transfer after substrate oxidation (Mo^IV^ → FAD, Fig. 2) is very fast, so NO_2_^–^ must react with the Mo^IV^ active site *before* its electrons are transferred to the other redox-active cofactors in the enzyme. Nitrite/hypoxanthine turnover was examined electrochemically at a glassy carbon/XO/glutaraldehyde electrode using the same procedure as above with methylene blue as the mediator, hypoxanthine as the reducing substrate and nitrite as the oxidizing substrate. In this case, nitrite and methylene blue are in competition for electrons derived from hypoxanthine oxidation. An expected rise in catalytic current upon addition of hypoxanthine (at a saturating level of 200 µM) is observed (Fig. 6A, red curve). Addition of nitrite (10 mM) leads to an immediate drop in catalytic current (Fig. 6A, yellow curve), which subsequently decreases further with successive cycling over about 40 min (grey curves). Replacement with a fresh buffer solution of methylene blue and hypoxanthine did not recover activity, which shows that XO has been inactivated. The only feasible candidates for such inactivation are NO_2_^–^ (substrate) or NO (product).

Nishino and co-workers reported that NO irreversibly inactivates XO and showed by EPR spectroscopy that the catalytically essential sulfido ligand is lost from the molybdenum centre when incubated with NO under reducing conditions [39]. This behaviour mimics the behaviour of cyanide which leads to the same desulfido form, with SCN^–^ as a product [40]. Likewise, this indicates that the product of nitrite reduction, NO, is responsible for the loss of XO (hypoxanthine oxidase) activity in Fig. 6A. Maia and Moura reported no loss of nitrite reductase XO activity during nitrite turnover and the origins of this disparity remain unclear [21, 22].

Although the loss of XO activity with time is apparent in Fig. 6A, the origin of this effect requires further investigation. XO contains 4 cofactors (Mo cofactor, two 2Fe-2S clusters and FAD) and all are essential for XO activity. The desulfido Mo cofactor may be restored to its active Mo=S state by redox cycling in the presence of sulfide ions [41]. An electrochemical adaptation of this protocol was developed, and 12 successive CV cycles were run (between − 200 and − 600 mV vs. SHE) with the glassy carbon/XO/glutaraldehyde electrode in a solution of methyl viologen (100 µM) and Na_2_S (10 mM) in HEPES buffer (pH 7); under these conditions the solution contains a mixture of HS^–^ and H_2_S. After electrode treatment, a CV of a fresh solution of methylene blue (10 µM) was run (Fig. 6B, broken curve). Addition of hypoxanthine (200 µM) again gave the anticipated catalytic current (Fig. 6B, solid curve) and XO activity was restored to approximately 80% of what was observed before nitrite addition (in Fig. 6A). These results clearly show that the loss of activity triggered by nitrite reduction at the Mo cofactor was due to loss of the catalytically essential Mo=S moiety.

## Conclusions

This study has demonstrated that an active and stable glassy carbon/XO/glutaraldehyde electrode is easily prepared and responds to both hypoxanthine and xanthine (using methylene blue as the mediator) in producing catalytic currents. These studies complement previous work on a bacterial xanthine dehydrogenase using phenazimum methosulfate as a mediator at a chemically modified gold electrode [26, 27, 34], although in these cases the enzyme-modified Au electrode required a membrane covering the surface to prevent enzyme dissociation into the bulk solution.

The role of XO as a potential nitrite reductase is not established and conflicting biochemical kinetic data have been reported that vary by orders of magnitude [13, 19, 21, 22, 42, 43]. In this work we have indirectly shown that XO in its (hypo)xanthine-reduced form can reduce nitrite but at the cost of time-dependent deactivation by the product NO which desulfurates the molybdenum centre. This was shown by XO activity being recovered through redox cycling in the presence of sulfide to regenerate the Mo=S form of the enzyme. These results challenge the physiological relevance of XO as a nitrite reductase as the decline in catalytic activity upon exposure to product NO through desulfuration cannot be reversed in vivo. The kinetics of XO deactivation by NO remains unknown. Such a study would require accurate concentrations of NO and a rapid method for measuring enzyme activity as a function of time, which our electrochemical method reported here may provide from the steady state catalytic current.

## Supplementary Information

Below is the link to the electronic supplementary material.

Supplementary Material 1

## Data Availability

All data are in the manuscript and supporting information.
