# Supplementary material for: Bovine xanthine oxidase electrocatalysis: substrate oxidation and its role in nitrite reduction
Source: J Biol Inorg Chem. 2026 Apr 27;31(3):145–51. doi: 10.1007/s00775-026-02146-z (PMC13287112; doi:10.1007/s00775-026-02146-z)
Supplement: Supplementary file 1 — Supplementary Material 1 [file 775_2026_2146_MOESM1_ESM.pdf]

Supporting Information

Bovine Xanthine Oxidase Electrocatalysis: substrate oxidation and its role in nitrite reduction

Peter D. Giang,[1] Dimitri Niks,[2] Russ Hille,[2] Paul V. Bernhardt[1]

Paul V. Bernhardt: p.bernhardt@uq.edu.au

[1] School of Chemistry and Molecular Biosciences, University of Queensland, Brisbane 4072, Australia
[2] Department of Biochemistry, University of California, Riverside, CA 92521, USA

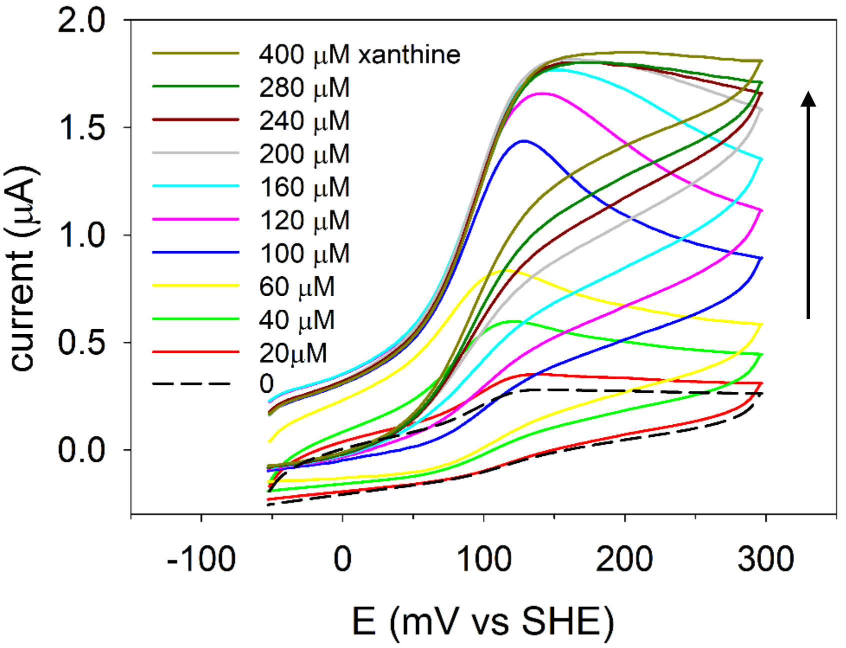

Figure S1. CVs of phenazine methosulfate (10 μM) at a glassy carbon/XO/glutaraldehyde electrode in the absence (broken curve) and presence of xanthine at the concentrations shown. Scan rate 5 mV s^{-1}, 200 mM HEPES buffer (pH 7).

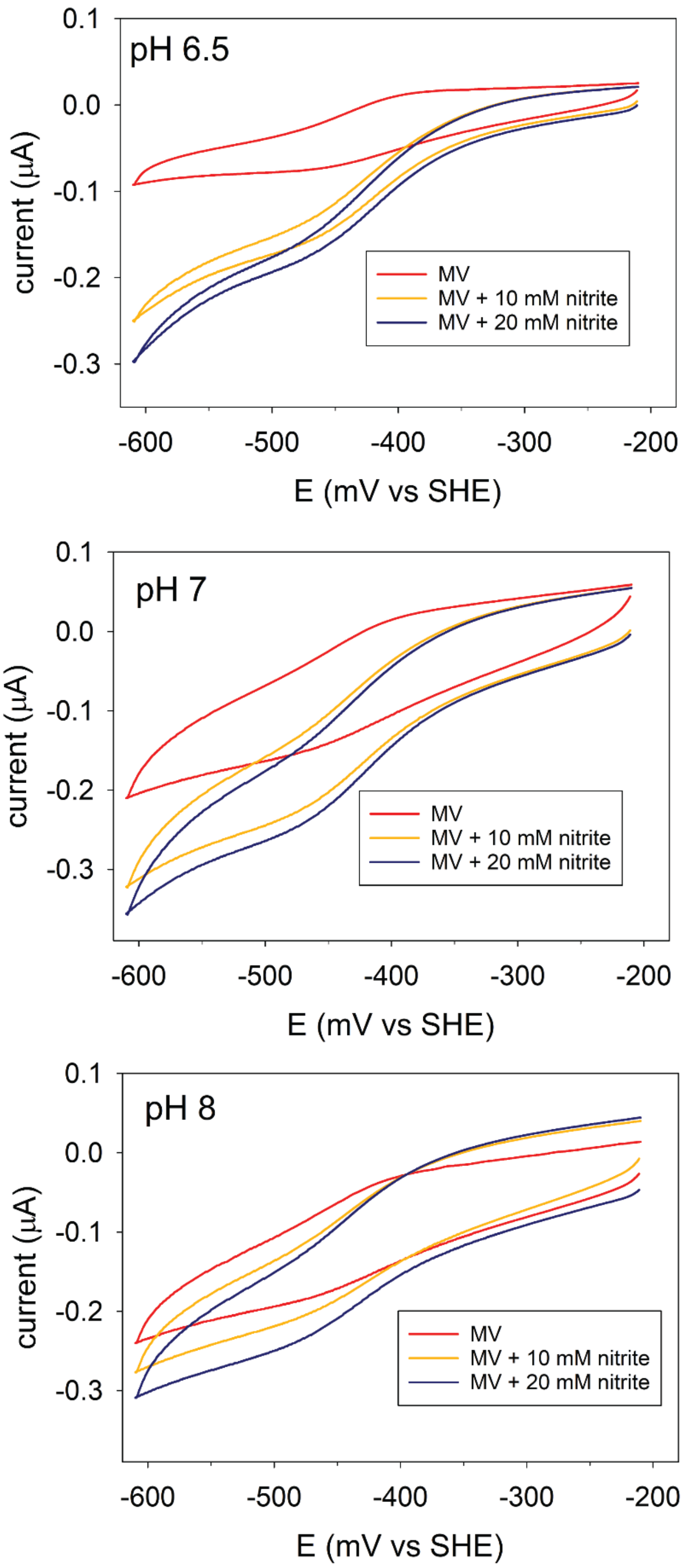

Figure S2. pH-dependent CVs (200 mM bis-tris acetate pH 6.5, 200 mM HEPES pH 7 and 8) of methyl viologen (20 μM) at a glassy carbon/glutaraldehyde electrode (no xanthine oxidase) in the absence (red curve) and presence of 10 mM nitrite (yellow) and 20 mM nitrite (blue). Scan rate 5 mV s^{-1}.

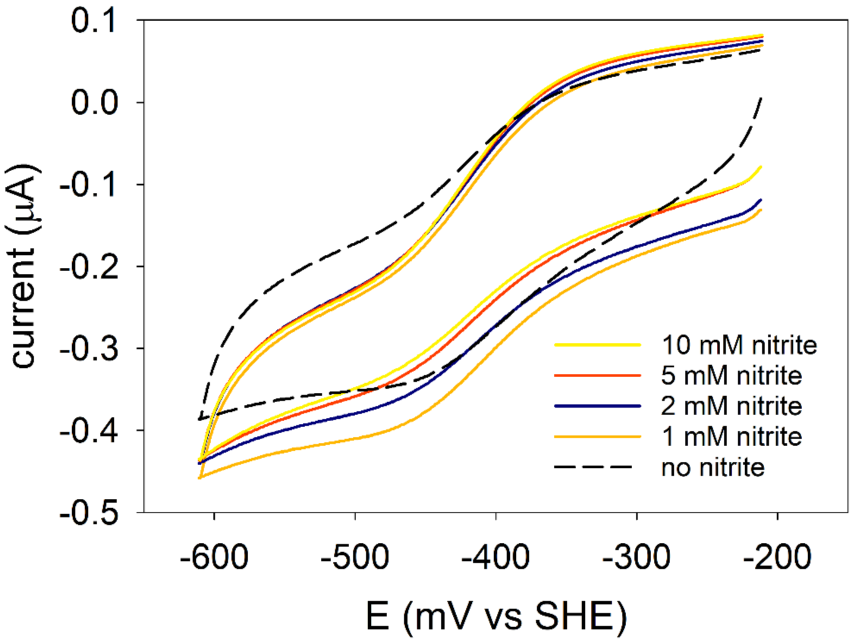

Figure S3. CVs of MV (20 μM) at a glassy carbon/XO/glutaraldehyde electrode in the absence (broken curve) and presence of nitrite (1 – 10 mM). Scan rate 5 mV s^{-1}, 200 mM Bis-tris acetate, pH 6.5.
